# *Escherichia coli* Antibiotic Resistance Patterns from Co-Grazing and Non-Co-Grazing Livestock and Wildlife Species from Two Farms in the Western Cape, South Africa

**DOI:** 10.3390/antibiotics10060618

**Published:** 2021-05-22

**Authors:** Michaela Sannettha van den Honert, Pieter Andries Gouws, Louwrens Christiaan Hoffman

**Affiliations:** 1 Centre for Food Safety, Department of Food Science, University of Stellenbosch, Private Bag X1, Matieland 7602, South Africa; michaelavdh@sun.ac.za; 2 Department of Animal Sciences, University of Stellenbosch, Private Bag X1, Matieland 7602, South Africa; louwrens.hoffman@uq.edu.au; 3 Centre for Nutrition and Food Sciences, Queensland Alliance for Agriculture and Food Innovation, The University of Queensland, Digital Agricultural Building, 8115, Office 110, Gatton 4343, Australia

**Keywords:** antimicrobial resistance, disc diffusion, game, ungulate, wildlife, livestock, cattle, sheep

## Abstract

Although limited, studies have found conflicting results on whether co-grazing results in significant antibiotic resistance transfer between species. This type of farming system can act as a vector in the geographical spread of antibiotic-resistant bacteria in the environment. The aim of this study was to determine the antibiotic-resistant patterns between co-grazing and non-co-grazing livestock and wildlife species in South Africa. *Escherichia coli* was isolated from the faeces of various wildlife and livestock species from two farms in South Africa and was tested for antibiotic resistance using the Kirby–Bauer disk diffusion method against chloramphenicol, nalidixic acid, ampicillin, streptomycin, sulphafurazole, and tetracycline. A selection of some common antibiotic-resistant genes (*bla*CMY, *aad*A1, *sul*1, *sul*2, *tet*A, and *tet*B) were detected using PCR. The *E. coli* isolates from wildlife and livestock that co-grazed showed no significant differences in antibiotic resistance patterns. However, this was not the case for tetracycline resistance as the livestock isolates were significantly more resistant than the co-grazing wildlife isolates. The *E. coli* isolates from the non-co-grazing livestock and wildlife had significant differences in their antibiotic susceptibility patterns; the wildlife *E. coli* isolates were significantly more resistant to sulphafurazole and streptomycin than the livestock isolates, whilst those isolated from the cattle were significantly more resistant to ampicillin than the wildlife and sheep isolates. The results of this study suggest that there could be an exchange of antibiotic-resistant bacteria and genes between livestock and wildlife that co-graze.

## 1. Introduction

Despite the perception of a low risk of antibiotic resistance developing in natural environments, the studies listed [1] have shown that antibiotic resistance among wild animals is a growing public health issue due to increased wildlife contact between humans, livestock, and domestic animals. It cannot be assumed that microbial communities in natural, more remote environments are completely isolated from external activities and commercial settings [2].

This study focused on the effect that co-grazing livestock and wildlife that live and feed off the same land may have on the development and transfer of antibiotic resistance between the wildlife–livestock interface. In South Africa, 34.3% of game farmers practised co-grazing of livestock and wildlife [3]. The wildlife–livestock interface is becoming a more common occurrence in animal farming, which is in part due to the increased demand for food and land as well as a direct response to re-wilding.

The transfer of antibiotic-resistant bacteria can be bi-directional at the wildlife–livestock interface, as both species can be sources of antibiotic-resistant bacteria [4,5,6]. There is evidence that transmission of microorganisms, whether it is antibiotic-resistant or disease-causing microorganisms, occurs between livestock and wildlife, demonstrated by numerous outbreaks of wildlife-associated diseases, such as Foot and Mouth disease and African swine fever, which have spread to domestic livestock [7]. It was found that 41% of farmers in South Africa who practised co-grazing of livestock and wildlife had no control measures to prevent animal interaction to prevent disease transmission [3]. This is a public health issue, as about 60% of diseases in humans are zoonotic, with 72% being of wildlife origin [8].

During co-grazing, direct contact can occur through interspecies contact during the sharing of pastures and water points [7,9] or indirectly, through mobile transfer vectors such as birds or wind [10,11]. Differences in contact rates can influence the transmission rate of diseases and bacteria between livestock and wildlife species. For example, in periods of drought, there is more frequent contact at existing water points [12]. These same principles regarding disease transfer also hold true for the exchange of antibiotic-resistant bacteria between co-grazing animals [12].

It was hypothesised that the practice of co-grazing wildlife and livestock influences the antibiotic resistance profiles of *Escherichia coli* harboured by animal species.

## 2. Results

### 2.1. Phenotypic Antibiotic Resistance

*Escherichia coli* was isolated from 35 of the 40 faecal samples. Metzler-Zebeli et al. [13] found that freezing animal faecal samples at −20 °C had a minimal effect (approximately a 3–6% loss) on the abundance of *Enterobacteriaceae* when compared to sampling directly from fresh faecal samples. *Escherichia coli* was selected for the analysis as it is commonly used as an indicator of antibiotic resistance by other researchers due to its ability to easily acquire and transfer antibiotic resistance genes and because *E. coli* is a commensal bacterium found in the normal gut flora of animals [14].

Overall, the *E. coli* isolates from livestock and wildlife species from both farms exhibited low resistance to the range of six antibiotics tested for. The *E. coli* isolated from the wildlife were, on average, 70% susceptible, and those isolated from the livestock were, on average, 70% susceptible to the six antibiotics.

It should be noted that the antibiotic resistance profiles of the *E. coli* isolates towards nalidixic acid and chloramphenicol were not included in the statistical analysis due to negligible resistance levels detected, leading to no variance in the data.

**Figure 1:**
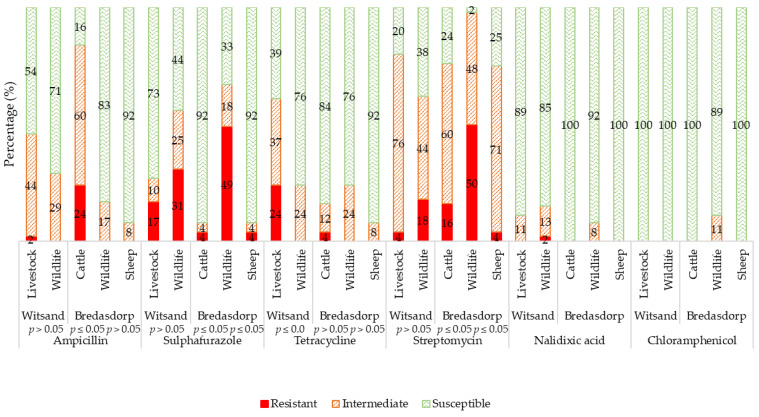
Antibiotic susceptibility patterns of *E. coli*-isolated sheep (*n* = 5, N = 24) and cattle (*n* = 5, N = 21) (livestock), and springbok (*n* = 1, N = 3) and deer (*n* = 4, N = 19) (wildlife) from a co-grazing farm (Witsand) and from sheep (*n* = 5, N = 24), cattle (*n* = 5, N = 25), eland (*n* = 5, N = 24) and wildebeest (*n* = 5, N = 22) (wildlife) from a non-co-grazing farm (Bredasdorp). *n* = number of animals tested, N = total number of isolates tested.

Figure 1 displays the overall antibiotic resistance levels of *E. coli* isolated from various animals from the Witsand farm (co-grazing farm) and the Bredasdorp farm (non-co-grazing). The springbok (*Antidorcas marsupialis*) and fallow deer (*Dama dama*) from the Witsand farm were grouped together as ‘wildlife’, and the sheep and cattle were grouped together as ‘livestock’ due to no differences (*p* > 0.05) in their antibiotic susceptibility profiles. Likewise, the eland (*Taurotragus oryx*) and wildebeest (*Connochaetes gnou*) from the Bredasdorp farm were grouped together as ‘wildlife’ due to no differences (*p* > 0.05) in their antibiotic susceptibility profiles. However, the sheep and cattle were grouped separately due to differences (*p* ≤ 0.05) in their antibiotic susceptibility profiles.

From the Witsand farm (co-grazing), on average, there were no differences (*p* > 0.05) in the antibiotic resistance levels between the co-grazing livestock and wildlife *E. coli* isolates except towards tetracycline, where the livestock isolates had a significantly higher number of isolates classified as resistant than the wildlife isolates (Figure 1).

From the Bredasdorp farm (no co-grazing), on average, there were differences (*p* ≤ 0.05) in the antibiotic resistance levels between the non-co-grazing livestock and wildlife except towards tetracycline. More specifically, the wildlife *E. coli* isolates were significantly more frequently classified as resistant to sulphafurazole and streptomycin than the cattle and sheep isolates, and the isolates from the cattle were significantly more frequently classified as resistant to ampicillin than the wildlife and sheep isolates (Figure 1).

### 2.2. Genotypic Antibiotic Resistance

The resistance genes selected for each corresponding antibiotic was selected based on some of the resistance genes that have been found to be the most common to *E. coli*; however, there are numerous antibiotic resistance genes, and only a small selection was used in this study. The following antibiotic-resistant genes were selected for testing: *bla*CMY gene (ampicillin resistance), *sul*1 and *sul*2 genes (sulphonamide resistance), *aad*A1 gene (streptomycin resistance), and *tet*A and *tet*B genes (tetracycline resistance). Resistant genes for chloramphenicol and nalidixic acid were not tested due to negligible phenotypic resistance levels.

In streptomycin-resistant *E. coli*, the *str*A–*str*B gene pair and the *aad*A gene cassette have been found to be the most common streptomycin-resistant genes [15,16]. There are six genes that encode for efflux pump proteins that have been identified in tetracycline-resistant *E. coli* strains, with the most common being *tet*A, *tet*B, and *tet*C [17]. The acquisition of altered target enzymes, which act as competitive inhibitors of dihydropteroate synthetase, known as dihydropteroate synthases, are the most common mechanism with which *E. coli* acquires resistance to sulphonamides [18,19]. There are three genes that encode for three types of these enzymes that have been characterised in Gram negatives, namely *sul*1, *sul*2, and *sul*3. Beta-lactam antibiotic resistance in *E. coli* is primarily mediated by the production of β-lactamase enzymes that inactivate the antibiotic [20]. Over 200 β-lactamases have been identified, of which the TEM-1, TEM-2 (*bla*TEM gene), CTX-M (*bla*CTX-M gene), SHV-1 (*bla*SHV gene), and CMY-2 (*bla*CMY-2 gene) enzymes are the most common in *E. coli* [20]. The *bla*TEM1 gene has been found to be the most common determinant observed in ampicillin-resistant *E. coli* of animal origin [21]. The *flo*R and *cml*A for *E. coli* chloramphenicol resistance and *gyr*A and *gyr*B, *par*C and *par*E, or *cat*1 and *cat*2 for nalidixic acid *E. coli* resistance are some of the most common antibiotic resistance genes [22,23,24]. These genes were not analysed in this study however, as no isolates showed phenotypic resistance to these two antibiotics. Table 1 shows the antibiotic resistance phenotype–genotype correlation of the *E. coli* isolates.

## 3. Discussion

### 3.1. Phenotypic Antibiotic Resistance

Antibiotic-resistant bacteria were found in both the livestock and wildlife faecal *E. coli* isolates, irrespective of whether they co-grazed (Figure 1). This suggests that antibiotic-resistant bacteria are present in natural environments, possibly originating from the natural reservoirs in the soil or even transferred from other nearby reservoirs, such as commercial farm effluent and transmitted to the natural environment by numerous vectors, such as birds, rodents, and rivers [25,26]. It has been found that antibiotic-resistant genes in the soil, produced by fungi and bacteria in the soil, such as *Actinobacteria*, are similar to those found in clinical settings in human pathogens, suggesting that one of the main originators of antibiotic resistance is, in fact, the environmental microbiota [10,27,28,29]. Other studies have also found antibiotic resistance in bacterial isolates from wildlife species and the natural environment, along with more frequent documentation of zoonotic disease infections, and suggest that wildlife could serve as a reservoir and transfer vector of antibiotic resistance of environmental origin [5,30,31].

All four wildlife species in this study were predominantly grazers with minimal browsing. The grazing/browsing nature of the game species analysed in this study may also play a part in the transfer and development of antibiotic resistance as grazing allows for more direct contact with the soil bacteria, which is said to contain naturally produced antimicrobial compounds and the accompanying antibiotic-resistant genes. King and Schmidt [5] revealed that the antibiotic resistance levels of bacteria from wildebeest and zebra (grazers) were higher than those from giraffe (browser), indicating the influence of soil bacteria on antibiotic resistance.

All of the *E. coli* isolates showed negligible resistance towards nalidixic acid, where 0.4% of isolates were classified as resistant to nalidixic acid and 8% were classified as intermediately resistant to nalidixic acid. Nalidixic acid was the first quinolone antibiotic used in animals but is no longer clinically used due to its toxicity, to resistance emergence, and to the development of more effective agents [32]. Thus, it can be hypothesised that there is no significant quinolone-selective pressure present in the farming environments of Witsand or Bredasdorp to promote resistance development. Costa et al. [33], Lillehaug et al. [34], and Silva et al. [6] reported similar low antibiotic resistance levels to nalidixic acid, ranging from 0 to 14% resistance in wild animals. Rolland et al. [35] also reported that nalidixic acid resistance was uncommon in the wildlife isolates.

All of the *E. coli* isolates showed negligible resistance towards chloramphenicol, where no isolates were classified as resistant to chloramphenicol and only 2% were classified as intermediately resistant to chloramphenicol. Chloramphenicol is prohibited for use in food-producing animals due to its severe side-effects in humans [36]. This suggests that chloramphenicol is not used on the Bredasdorp and Witsand farms and their surroundings as no selective pressure is evident. The very low resistance in this study is consistent with other studies that detected 0–7% resistance to chloramphenicol in various wild animals and reported that resistance to chloramphenicol is rare [6,33,34,35].

Figure 1 shows that the *E. coli* isolates from the livestock and wildlife species had the highest resistance towards streptomycin, with only 13% showing susceptibility to this antibiotic. The high level of resistance to streptomycin was expected because the wildlife and livestock species, due to their grazing nature, can easily pick up streptomycin-resistant bacteria and streptomycin-resistant genes that are naturally present in the soil, produced by *Actinobacteria* [37]. Furthermore, streptomycin is one of the most used antibiotics in agriculture, in use since 1936. Resistance has become prevalent worldwide, where resistant clinical isolates have been evident since the late 1940s [38].

Resistance to ampicillin was the second highest from the faecal samples, where 69% of isolates were susceptible. The significantly higher percentage of resistance towards ampicillin from the cattle isolates from the Bredasdorp farm (non-co-grazing) could have originated from the frequent application of penicillin to treat diseases (Figure 1). Penicillin is one of the most commonly used antibiotic classes in food-producing animal farming [39]. The high ampicillin resistance of the isolates from the cattle was not transferred to the sheep or wildlife.

On average, the *E. coli* isolates were 47% susceptible to sulphafurazole, a synthetic antibiotic. On both farms, the wildlife isolates showed significantly higher resistance (31% and 49%) to sulphafurazole than the livestock isolates (17% and 4%). Other studies have also found *E. coli* isolates originating from natural environments to be highly resistant to sulphonamides due to the presence of a naturally occurring resistant enzyme [40]. Many indirect factors, such as the presence of heavy metals, can also be a causative factor for the onset of sulphonamide-resistant bacteria in nature [41]. Sulphonamide-resistant bacteria could also find their way into the wildlife and livestock territory via indirect pathways, such as water run-off or the application of manure from intensive farming that can be dissipated into the ground, as resistance to sulphonamides is common in intensively reared farm animals [25].

On average, there was a low level of resistance towards tetracycline, except from the *E. coli* isolates originating from the livestock from the Witsand farm (24% resistant). This resistance was not carried over to the co-grazing wildlife. Tetracycline is also a commonly used antibiotic in livestock farming, used to treat various infections and for growth promotion [42,43,44]. This level of resistance could also be due to tetracycline being used to treat diseases of the livestock on the Witsand farm.

On the Bredasdorp farm (no co-grazing), the only nonsignificant difference in the antibiotic resistance profiles between the non-co-grazing livestock and wildlife *E. coli* isolates was towards tetracycline. This is due to an overall low level of antibiotic resistance in both the livestock (4%) and wildlife (0%) *E. coli* isolates, leading to little variance in the data. On the contrary, on the Witsand farm (co-grazing), the only significant difference in the antibiotic resistance profiles between the co-grazing livestock (24% resistant) and wildlife (0% resistant) *E. coli* isolates was towards tetracycline. More specifically, the tetracycline resistance originated from one sheep and three cattle. This resistance pattern could be attributed to the fact that the livestock were occasionally treated therapeutically with tetracycline as required.

### 3.2. Genotypic Antibiotic Resistance

There were a few genotypic results that did not agree with the phenotypic results where the antibiotic-resistant gene was detected but the phenotypic method classified the isolate as susceptible. Antibiotic resistance genes were detected in seven of the ampicillin susceptible isolates and two of the tetracycline susceptible isolates. This discrepancy could be due to these antibiotic-resistant genes being inactive in the host, as there would be no significant antibiotic selection pressure in these more remote areas of no to low antibiotic use to warrant these genes being active or “switched on” [45].

There were also a few cases where none of the selected antibiotic-resistant genes were detected but the isolate were shown to be phenotypically resistant. This was the case in two of the sulphafurazole-resistant isolates and two of the tetracycline-resistant isolates. These discrepancies are likely due to the isolate possessing a different antibiotic-resistant gene, which was detected in this study, as there are many different antibiotic resistance genes that can confer the same phenotypic antibiotic resistance pattern. These discrepancies highlight the importance of combining genotypic and phenotypic methods for determining the antibiotic resistance patterns of bacterial species.

Katakweba et al. [46] and Navarro-Gonzalez et al. [47] did not find any significant differences in the antibiotic resistance profiles of bacteria between co-grazing wildlife and wildlife species that were isolated (non-co-grazing). In contrast, Mercat et al. [9] found that wildlife at the interface with livestock had higher resistance levels than those with no contact with livestock, suggesting a transfer in antibiotic resistance through co-grazing. These disputed points highlight the complexity of antibiotic resistance and transfer in nature, a highly complex ecosystem.

## 4. Materials and Methods

### 4.1. Study Area and Sample Collection

Springbok, fallow deer, Angus cattle, and Merino sheep faecal samples were collected from a farm in Witsand, Western Cape, South Africa. The wildlife species were free to co-graze with the livestock species. Both the livestock and wildlife grazed and drank (natural streams and ground/borehole water) on the farm’s natural resources, although in times of drought, the livestock were supplied with supplementary feed. The supplementary feed did not contain any antibiotics. This farm is a commercial free-range farm where the livestock are sold for slaughter and the wildlife are only slaughtered for personal use. The livestock are given penicillin or tetracycline as required to treat diseases. The wildlife do not receive any medication.

Eland, black wildebeest, Angus cattle, and Merino sheep faecal samples were collected from a farm in Bredasdorp, Western Cape, South Africa. The wildlife species were separated from the livestock by a fenced off region that did not allow any wildlife to leave the fenced off area. The wildlife grazed on pastures and drank from the farm’s dam. The livestock were fed a supplementary feed daily and drank from the ground/borehole water, which was pumped into drinking troughs. The supplementary feed did not contain any antibiotics. This farm is also a commercial free-range farm where the livestock are sold live or slaughtered and the wildlife are infrequently sold live for breeding. Similar to the Witsand farm, the livestock on the Bredasdorp farm also receive penicillin or tetracycline as required to treat diseases. The wildlife also do not receive any medication.

A summary of the sample species, farm location, type of farm, and number of samples collected is shown in Table 2. All four of the wildlife species are ungulates and are all grazers or mixed grazers-browsers.

Livestock faecal (≈20 g) samples were collected from the ground shortly after deposition into sterile sample containers. All of samples from the same farm were collected during the same time period on the same day to avoid possible repetitive sampling.

Wildlife faecal (≈20 g) samples were aseptically collected in a sterile container from the middle of the small intestine after evisceration from recently slaughtered animals. All of collected samples were then transported on ice at ≈4 °C to the university’s laboratory freezer (−20 °C) within 24 h of sampling. Before analysis commenced, the samples were defrosted at room temperature.

### 4.2. Isolation of Escherichia coli

Approximately 90 mL of buffered peptone water (Merck Biolab, Modderfontein, South Africa) and 10 g of faecal matter was aseptically added into a stomacher bag and homogenised for 2 min using a stomacher device. The sample was then incubated at 37 °C for 12–14 h. This resuscitation step allows for better recovery of bacterial cells due to frozen storage [48].

The pour plate technique was used, using a violet red bile dextrose agar (VRBDA) (Merck Biolab, Modderfontein, South Africa) and 10^−4^ and 10^−5^ dilutions of the original sample. These dilutions were found to produce single colonies in the range of 25 to 250 colonies per plate, thus allowing single colonies to be easily obtained. The plates were inverted and incubated overnight at 37 °C.

Characteristic colonies from the VRBDA plates were streaked onto an eosin methylene blue agar (Oxoid, Hampshire, UK). The plates were inverted and incubated overnight at 37 °C.

Five presumptive *E. coli* colonies were selected per animal species, and their identities were confirmed using Gram’s stain and the citrate utilisation test using Simmons citrate agar (Oxoid, Hampshire, UK). Glycerol stock cultures were made of the confirmed *E. coli* isolates.

### 4.3. Antibiotic Susceptibility Testing

The Kirby–Bauer disk diffusion method was used according to the Clinical and Laboratory Standards Institute (CLSI) 2018 guidelines, using fresh overnight cultures grown on nutrient agars (Merck Biolab, Modderfontein, South Africa). A quality control strain, *E. coli* ATCC 25922 (Thermo Fisher Scientific, Lake Charles, LA, USA), and a negative control plate were used.

The antibiotic discs (Oxoid, Johannesburg, South Africa) containing ampicillin (10 μg), chloramphenicol (30 μg), nalidixic acid (30 μg), streptomycin (10 μg), sulphafurazole (300 μg), and tetracycline (30 μg) were placed on the inoculated Mueller–Hinton agar (Merck Biolab, Modderfontein, South Africa) plates using an automatic six-disc dispenser (Oxoid, Johannesburg, South Africa).

The selection of antibiotics were chosen based on those that are commonly used in the animal farming sector in South Africa as well as nalidixic acid and chloramphenicol, as these two antibiotics are no longer used, where the latter has been banned for use in animal farming.

The bacteria’s antibiotic susceptibility profile was determined using the diameter of the zone of inhibition and the parameters published by the CLSI 2018. The disc diffusion test was performed on five *E. coli* isolates that were isolated from each animal.

### 4.4. Statistical Analysis

The categorised results were analysed using one-way analysis of variance (ANOVA) using the Statistica 13.2 software (Tibco, Palo Alto, CA, USA). Levene’s test was applied to determine homogeneity of variance. The main effect was the practice of co-grazing and non-co-grazing. If the group means were significantly different within the groups, post hoc tests were performed to determine where the differences occurred within each group. Significant results were identified by least significant means (LSM) with a 95% confidence interval.

### 4.5. Gene Detection

DNA from fresh overnight cultures was extracted using a crude extraction method involving boiling and lysis buffer. The extracted DNA concentration and quality were established using a Nanodrop- 1000 spectrophotometer (Thermo Fisher Scientific, Wilmington, DE, USA) before PCR commenced.

Polymerase chain reaction (PCR) was used to detect a selection of antibiotic-resistant genes in a representative selection of isolates showing phenotypic resistance. The genes selected and the primers and reaction conditions are listed in Table 3. The reactions were performed in duplicate. Each reaction (25 µL) consisted of 1 unit of Ampliqon multiplex TEMPase 2× Master Mix (Ampliqon, Ondense, Denmark), 1 µL of template DNA, 0.2 µM each of the forward and reverse primers (Inqaba Biotec, Muckleneuk, South Africa), and the remaining volume distilled nuclease-free water (Inqaba Biotec, Muckleneuk, South Africa).

The PCR product was applied to a 1.2% agarose gel (Lonza SeaKem, Rockland, ME, USA) stained with EZ-Vision^®^ in-gel solution DNA dye (Amresco, Solon, OH, USA), using electrophoresis run at 85 V for 60–90 min and then visualized using the Bio-Rad Gel Doc XR + System (Bio-Rad, Hercules, CA, USA) in combination with Image Lab Software V5.2.1. A 100 bp DNA ladder was used (New England BioLabs Inc., Ipswich, MA, USA) as a reference marker.

## 5. Conclusions

Although the overall results of this study suggest that, on these two farms in the Western Cape (South Africa), co-grazing may result in a transfer of antibiotic resistance bacteria and genetic elements between co-grazing wildlife and livestock and that separating livestock and wildlife may help to reduce this transfer, this remains a complex phenomenon, which warrants further investigation.

## 6. Limitations of Study

This study was limited by the fact that neither the livestock nor wildlife isolates had significantly high antibiotic resistance levels, lessening the potential effect of transmission within the livestock–wildlife interface. This study was also limited in the number of samples and farms used, in the number of antibiotics used for the analysis, and in the number of antibiotic resistance genes detected. Therefore, additional studies are recommended to confirm whether the practice of co-grazing can lead to the transfer and exchange of antibiotic resistance and, potentially, shared pathogens and diseases.

## Figures and Tables

**Table 1:** Correlation between *E. coli* phenotypic antibiotic resistance and PCR results.

Farm	Animal	Phenotypic Resistance ^1^	Genotypic Resistance					
			*bla*CMY	*sul*1	*sul*2	*aad*A1	*tet*A	*tet*B
Bredasdorp	Cattle 1	AMP(I), SF(S), ST(R), TE(S)	-	-	-	+	-	-
Bredasdorp	Cattle 2	AMP(I), SF(S), ST(I), TE(S)	-	-	-	+	-	-
Bredasdorp	Cattle 3	AMP(R), SF(S), ST(I), TE(S)	+	-	-	-	-	-
Bredasdorp	Sheep 1	AMP(S), SF(S), ST(R), TE(I)	-	-	-	+	-	-
Bredasdorp	Sheep 1	AMP(S), SF(S), ST(I), TE(S)	+	-	-	+	-	-
Bredasdorp	Eland 1	AMP(S), SF(S), ST(R), TE(S)	+	-	-	+	-	-
Bredasdorp	Eland 1	AMP(S), SF(R), ST(I), TE (S)	-	-	+	+	-	-
Bredasdorp	Eland 1	AMP(S), SF(S), ST(R), TE(I)	-	-	-	+	+	-
Bredasdorp	Wildebeest 1	AMP(S), SF(S), ST(R), TE(S)	+	-	-	+	-	-
Bredasdorp	Wildebeest 2	AMP(S), SF(R), ST(R), TE(I)	-	-	+	+	-	-
Bredasdorp	Wildebeest 3	AMP(S), SF(R), ST(I), TE(S)	+	-	+	-	+	+
Bredasdorp	Wildebeest 4	AMP(S), SF(S), ST(R), TE(I)	+	-	-	+	-	-
Witsand	Cattle 1	AMP(I), SF(S), ST(I), TE(R)	-	-	-	+	+	+
Witsand	Cattle 2	AMP(I), SF(R), ST(R), TE(I)	-	-	-	+	-	-
Witsand	Sheep 1	AMP(R), SF(S), ST(I), TE(I)	+	-	-	+	-	-
Witsand	Sheep 2	AMP(I), SF(S), ST(I), TE(R)	-	-	-	+	-	-
Witsand	Deer 1	AMP(I), SF(S), ST(R), TE(S)	-	-	-	+	-	-
Witsand	Deer 2	AMP(S), SF(R), ST(R), TE(R)	-	+	+	+	-	+
Witsand	Deer 3	AMP(I), SF(R), ST(R), TE(R)	-	+	+	+	-	+
Witsand	Springbok 1	AMP(S), SF(S), ST(R), TE(S)	+	-	-	+	-	-
Witsand	Springbok 2	AMP(S), SF(S), ST(I), TE(S)	+	-	-	+	-	-

^1^ AMP, ampicillin (*bla*CMY gene); SF, sulphonamide (*sul*1 and *sul*2 genes); ST, streptomycin (*aad*A1 gene); TE, tetracycline (*tet*A and *tet*B genes); S, susceptible; I, intermediate; R, resistant.

**Table 2:** Details of the wildlife and livestock samples used in this study.

Wildlife Species	Farm Location	Farm Type	Number of Faecal Samples
Springbok (*Antidorcas marsupialis*)	Witsand	Co-grazing	5
Fallow deer (*Dama dama*)	Witsand	Co-grazing	5
Sheep (*Ovis aries*)	Witsand	Co-grazing	5
Cattle (*Bos taurus*)	Witsand	Co-grazing	5
Eland (*Taurotragus oryx*)	Bredasdorp	No co-grazing	5
Black wildebeest (*Connochaetes gnou*)	Bredasdorp	No co-grazing	5
Sheep (*Ovis aries*)	Bredasdorp	No co-grazing	5
Cattle (*Bos taurus*)	Bredasdorp	No co-grazing	5

**Table 3:** PCR conditions for detection of the selected antibiotic resistance genes.

Antibiotic	Gene	Primers F: 5′-3′ Primers R: 5′-3′	bp	Reaction Conditions	Reference
Tetracycline	*tet*A	F: GGCGGTCTTCTTCATCATGC R: CGGCAGGCAGAGCAAGTAGA	502	15 min at 95 °C;35 cycles of: 20 s at 95 °C, 40 s at 66 °C, 40 s at 72 °C; 4 min at 72 °C.	Adapted from [15]
	*tet*B	F: CATTAATAGGCGCATCGCTG R: TGAAGGTCATCGATAGCAGG	930		
Sulphafurazole	*sul*1	F: CGGCGTGGGCTACCTGAACG R: GCCGATCGCGTGAAGTTCCG	433	15 min 95 °C;30 cycles of: 20 s at 95 °C, 40 s at 66 °C, 40 s at 72 °C; 4 min at 72 °C.	Adapted from [25]
	*sul*2	F: CGGCATCGTCAACATAACCT R: TGTGCGGATGAAGTCAGCTC	721		
Ampicillin	*bla*CMY	F: GACAGCCTCTTTCTCCACA R: TGGACACGAAGGCTACGTA	1000	15 min at 94 °C;30 cycles of: 1 min at 94 °C, 1 min at 55 °C, 1 min at 72 °C; 10 min at 72 °C.	[25]
Streptomycin	*aad*A1	F: GTGGATGGCGGCCTGAAGCC R: AATGCCCAGTCGGCAGCG	525	15 min at 95 °C;35 cycles of: 1 min at 94 °C, 1 min at 60 °C, 1 min at 72 °C; 7 min at 72 °C.	Adapted from [15]

## Data Availability

The raw data presented in this study are available upon request to the corresponding author.
